# Outcomes of Coronary Artery Bypass Grafting in Patients With ST-Elevation Myocardial Infarction and the Impact of Impella: A Retrospective Single-Center Study

**DOI:** 10.7759/cureus.88394

**Published:** 2025-07-20

**Authors:** Kazuyoshi Takagi, Takahiro Shojima, Takanori Kono, Yasuyuki Zaima, Kosuke Saku, Maki Otsuka, Tatushiro Shibata, Toshiyuki Yanai, Yoshihisa Matsushima, Aya Nishikido, Miyu Hayashida, Rei Yamakawa, Hiroki Mine, Yusuke Shintani, Hiroyuki Otsuka, Tohru Takaseya, Koichi Arinaga, Yoshihiro Fukumoto, Eiki Tayama

**Affiliations:** 1 Division of Cardiovascular Surgery, Department of Surgery, Kurume University, Kurume, JPN; 2 Division of Cardiovascular Medicine, Department of Internal Medicine, Kurume University, Kurume, JPN

**Keywords:** coronary artery bypass grafting, impella, institutional outcome, percutaneous coronary intervention, st-elevation myocardial infarction

## Abstract

Objective: ST-segment elevation myocardial infarction (STEMI) often requires urgent revascularization, with percutaneous coronary intervention (PCI) as the primary strategy. However, coronary artery bypass grafting (CABG) remains essential in cases where PCI is either unfeasible or fails. Emergency CABG for STEMI is associated with high operative risk and poor outcomes, especially in hemodynamically unstable patients. The Impella device (Abiomed Inc., Danvers, MA) provides percutaneous mechanical circulatory support (MCS), which stabilizes hemodynamics, enhances end-organ perfusion, and facilitates myocardial recovery through left ventricular unloading. These effects may allow for clinical stabilization before surgery, thereby reducing the need for emergency CABG and improving surgical outcomes. This study aimed to evaluate the outcomes of CABG for STEMI and to assess how the introduction of Impella influenced its indications, timing, and patient selection.

Methods: We conducted a retrospective analysis of 59 consecutive patients with STEMI who underwent CABG between 2012 and 2023. Clinical data were obtained from the institutional electronic medical records for all patients included in this study. Patients were classified as requiring emergency, urgent, or elective procedures. Clinical and procedural data were compared before and after the introduction of Impella in 2019.

Results: The hospital mortality occurred in one patient (1.7%), including one patient (3.3%) among emergent cases. Postoperative complications occurred in 13 patients (43.3%) of emergency cases, five patients (27.8%) of urgent cases, and none of the elective cases. Sepsis and acute kidney injury were the most common complications. The length of stay in the intensive care unit was significantly longer for emergent cases than for urgent and elective cases. CABG for STEMI was performed in 49 cases before the introduction of Impella and in 10 cases afterward. This led to a decrease in the rate of CABG performed for STEMI from 11.2% to 4.3%. Although the difference was not statistically significant, the proportion of emergency CABG decreased from 53.1% to 40%, while urgent CABG increased from 26.5% to 50%, suggesting a trend toward surgical stabilization. Regarding the use of MCS, the rate increased from 53.1% (26 patients) before Impella introduction to 70% (seven patients) after, with Impella used in 20% (two patients) of the latter group.

Conclusion: CABG remains a viable option for patients with high-risk STEMI. Observations suggest that the use of Impella may be linked to a shift in CABG indications, from unsuitable PCI to failed PCI, and a tendency toward less emergent surgical timing.

## Introduction

ST-segment elevation myocardial infarction (STEMI) is a life-threatening condition that requires urgent revascularization to restore myocardial perfusion and minimize infarct size. Although percutaneous coronary intervention (PCI) remains the primary reperfusion strategy, coronary artery bypass grafting (CABG) is indicated in specific clinical scenarios where PCI is either infeasible or suboptimal. Emergency CABG is recommended for patients with ongoing ischemia or hemodynamic instability when PCI is not technically feasible. Conversely, elective CABG is typically reserved for patients with STEMI having stable hemodynamics who present with complex multivessel disease (MVD) or left main coronary artery (LMTD) involvement, particularly when durable revascularization is required [1,2]. Data from United States databases indicate a decline in the rate of CABG performed for STEMI, decreasing from 8.3% in 2007 to 5.4% in 2014, coinciding with advancements in PCI techniques [3]. In the context of STEMI complicated by cardiogenic shock (CS), PCI use increased from 34.9% in 2002 to 50.7% in 2014, whereas CABG use slightly declined from 14.2% to 13.9% during the same period [4].

The 30-day mortality rate for CABG in patients with acute myocardial infarction (AMI) ranges from 3% to 8% [5-8]. Risk factors associated with higher mortality include advanced age, renal dysfunction, reduced left ventricular ejection fraction (LVEF), CS, and diabetes [5,6,8]. Surgery performed within 24 hours of onset is associated with significantly higher mortality rates, reported to be between 8.4% and 19%, emphasizing the critical role of surgical timing [5,6,8]. However, CABG has been associated with better outcomes than PCI for AMI complicated by CS (AMI-CS), with lower in-hospital mortality rates (19% for CABG vs. 27% for PCI) [4,9]. Notably, 30% of CABG cases for STEMI are complicated by CS, and the disparity between the risks and benefits of CABG in such high-risk cases remains unresolved [7].

The Impella device (Abiomed Inc., Danvers, MA) is a percutaneous mechanical circulatory support (MCS) device designed to reduce left ventricular (LV) end-diastolic pressure and increase mean arterial pressure. These effects enhance coronary blood flow, promote myocardial recovery through LV unloading, and improve the perfusion of major organs. The Impella device has transformed the treatment of CS, contributing to a decline in the use of intra-aortic balloon pumps (IABPs) and a rapid increase in Impella cardiac power (CP) adoption in Western countries [10,11]. In addition, in the context of AMI, including STEMI, the use of Impella prior to reperfusion reduces infarct size [12,13]. The Danish‑German Cardiogenic Shock trial demonstrated that Impella CP use in AMI-CS reduced 180-day mortality compared to standard therapy, decreasing all-cause mortality by 13% [14]. In Japan, the 30-day survival rate of AMI-CS, previously reported to be 66.5% [15], improved to 80.9% following the introduction of the Impella device [16]. Beyond CS, Impella has shown utility in high-risk PCI cases, such as those with low LVEF, LMTD, or MVD [17,18].

Because the outcomes of CABG for STEMI are significantly influenced by the urgency of surgery and the presence of CS, this study first aimed to evaluate surgical outcomes across the full spectrum of urgency-emergency, urgent, and elective, as well as in patients with and without CS. Considering recent advances and given the potential of the Impella device to influence future STEMI treatment strategies, we further examined its impact on CABG indications, timing, and perioperative management in this high-risk population.

## Materials and methods

Study design

This was a single-center, retrospective observational study conducted at Kurume University Hospital, a tertiary emergency medical institution. The study aimed to evaluate the outcomes of CABG in patients presenting with STEMI and to examine the impact of the Impella device on case volume and treatment strategy. In particular, a comparative analysis was performed between cases before and after the introduction of the Impella device in April 2019. No patients were excluded from the study cohort. Adjustment for confounding variables was not performed.

Study population and sample size

The study included 59 consecutive patients with STEMI who underwent CABG between January 2012 and December 2023 at Kurume University Hospital. Patients were eligible if they were diagnosed with STEMI and subsequently treated with CABG during the study period. The cohort included patients treated at a facility where early reperfusion using PCI is the primary strategy for STEMI. CABG was performed in cases judged by the heart team to be unsuitable for PCI or where PCI had failed. In this study, the “unsuitable PCI” group was defined as patients in whom PCI was deemed inappropriate before the procedure and therefore not attempted.

The decision-making process involved a heart team consisting of staff from the cardiac care unit at the Advanced Critical Care and Emergency Center, interventional cardiologists, and cardiac surgeons. Surgical urgency was categorized as follows: 1) emergent: surgery performed immediately after the indication was established; 2) urgent: surgery performed within 72 hours of the indication; and 3) elective: surgery performed more than 72 hours after the indication.

Patients with progressive myocardial ischemia underwent immediate (emergent) CABG, while those stabilized by medical therapy or MCS, or those requiring management for acute heart failure, underwent urgent or elective CABG.

Surgical procedures

Standard cardiac surgical protocols were followed. CABG was performed through a full median sternotomy. The majority of cases used cardiopulmonary bypass (CPB) with cardiac arrest, moderate hypothermia (30℃-32℃), and cold crystalloid cardioplegia administered via an antegrade route. In three patients who received pre- or intraoperative Impella support, CABG was performed on a beating heart.

The left internal mammary artery (LIMA) was harvested in a skeletonized fashion and primarily grafted to the left anterior descending artery (LAD), unless hemodynamic instability necessitated a deviation from this strategy. The great saphenous vein was harvested using an open technique.

For patients undergoing beating heart CABG with Impella support, the device was set to maintain a mean arterial pressure of approximately 60 mmHg and a flow rate of 2-2.5 L/minute. Mixed venous oxygen saturation was maintained at ≥50%, and within −10%-20% of the preoperative baseline. These intraoperative targets for hemodynamic support were determined according to the standardized protocol of our department. Postoperative management in the intensive care unit (ICU) was standardized across all cases.

Study measures and statistical analysis

Preoperative, intraoperative, and postoperative clinical variables were analyzed to evaluate the surgical outcomes of CABG in STEMI patients. Preoperative echocardiographic data were unavailable in six patients (five emergency and one urgent), and postoperative data were missing in seven patients (five emergency and two elective). The primary endpoint was clinical status at hospital discharge. Major postoperative complications were defined as stroke, sepsis, pneumonia, deep sternal wound infection, acute kidney injury (AKI), initiation of new renal replacement therapy (RRT), reexploration for bleeding, and gastrointestinal bleeding. Additionally, the impact of the introduction of the Impella device in April 2019 on CABG case volume and surgical indications was assessed.

Data were extracted retrospectively from hospital records, including clinical notes, laboratory findings, echocardiographic results, and procedural details. All 59 patients were included in the analysis, and no exclusion criteria were applied. Confounding factors were not adjusted for in the statistical analysis.

Continuous variables were analyzed using the Kruskal-Wallis test. When a significant difference was found using the Kruskal-Wallis test, post hoc comparisons between the groups were performed using pairwise tests with appropriate adjustment for multiple comparisons. Categorical variables were compared using the chi-square test. A two-tailed p value of <0.05 was considered statistically significant. All statistical analyses were performed using the JMP® statistical software (SAS Institute Inc., Cary, NC).

Ethics statement

This study was conducted in accordance with the Declaration of Helsinki (revised in 2013). Ethical approval was obtained from the Institutional Review Board (IRB) of Kurume University (IRB no. 21001) on April 20, 2021. For cases treated before IRB approval, informed consent for retrospective data use was obtained individually or through opt-out procedures, as approved by the IRB.

## Results

Preoperative patient characteristics

The mean age of the patients was 69.8 ± 9.5 (range, 42-85) years. The mean body mass index was 23.6 ± 3.5 kg/m^2^. Among the risk factors for AMI, approximately 80% of the patients had hypertension, over 60% had dyslipidemia, and approximately 50% had diabetes, chronic kidney disease, or a history of smoking. The mean glycated hemoglobin A1c level was 6.9 ± 1.7 (range, 5%-11.9 %).

Overall, out-of-hospital cardiac arrest occurred in 13 patients (22%), and CS was present in half of all patients. The mean serum levels of creatine kinase (CK) and CK-MB at the time of admission were 887.1 ± 1,297.8 U/L (maximum: 7,235 U/L) and 68.3 ± 82.4 U/L (maximum: 378 U/L), respectively. The mean LVEF was 46%, with 16 patients (27.1%) having an LVEF of ≤35%. Coronary lesions involved the left main trunk with or without other vessel disease in 26 patients (44.1%), and triple-vessel disease in 22 patients (37.3%). Preoperative MCS was used in a mutually exclusive manner: IABP in 28 patients (47.4%), IABP with ECMO in three (5.1%), and Impella CP in two (3.4%). Preoperative patient characteristics are listed in Table 1.

**Table 1:** Preoperative patients’ characteristics PCI, percutaneous coronary intervention; SD, standard deviation; eGFR, estimated glomerular filtration rate; HbA1c, hemoglobin A1c; CK, creatine kinase; STEMI, ST-elevation myocardial infarction; LVEF, left ventricular ejection fraction; LMT, left main trunk; +α, with or without other vessel disease; MCS, mechanical circulatory assist device; IABP, intra-aortic balloon pumping; ECMO, extracorporeal membrane oxygenation

Variables	Values
Demographics	
Age (years)	69.8 ± 9.5 (range: 42-85)
Male, n (%)	48 (81.4%)
Body mass index (kg/m^2^)	23.6 ± 3.5
Comorbidities and past histories, n (%)	
Hypertension	46 (78%)
Diabetes	30 (50.8%)
Dyslipidemia	39 (66.1%)
Chronic kidney disease	25 (42.4%)
Hemodialysis	4 (6.8%)
Previous PCI	15 (25.4%)
Previous stroke	7 (11.9%)
Smoking history	27 (45.8%)
Peripheral artery disease	8 (13.6%)
Laboratory results, mean ± SD	
Hemoglobin (g/dL)	12.9 ± 2
eGFR (mL/minute/1.37 m^2^)	65.3 ± 25.5
HbA1c (%)	6.9 ± 1.7 (range: 5-11.9)
Presentation characteristics of STEMI	
Out-of-hospital cardiac arrest, n (%)	13 (22%)
Cardiogenic shock, n (%)	29 (49.2%)
CK (U/L), mean ± SD	887.1 ± 1,297.8 (max: 7,235)
CK-MB (U/L), mean ± SD	68.3 ± 82.4 (max: 378)
LVEF < 35%, n (%)	16 (27.1%)
Coronary angiography, n (%)	
LMT+α	26 (44.1%)
Three-vessel disease	22 (37.3%)
Two-vessel disease	10 (16.9%)
One-vessel disease	1 (1.7%)
Preoperative MCS, n (%)	
IABP	28 (47.4%)
IABP+ECMO	3 (5.1%)
Impella CP	2 (3.4%)
None	26 (44.1%)

Intraoperative and postoperative details

Emergency surgeries accounted for 30 patients (50%), whereas urgent surgeries comprised 18 patients (30%). According to the Japan score, predicted 30-day mortality increased with surgical urgency: 13.9% for emergency surgery, 8.7% for urgent surgery, and 4.3% for elective surgery. Predicted mortality was significantly higher in emergency than in elective cases (p < 0.05). The LIMA to LAD anastomosis was successfully performed in all urgent and elective surgeries. However, its use in emergency surgeries was limited to 21 patients (70%), primarily due to hemodynamic instability. The LIMA usage rate in emergency cases was significantly lower than in elective cases (p < 0.05). In addition, emergency surgeries typically involved fewer bypass grafts, necessitating more frequent postoperative MCS (p < 0.05). Among the emergency cases, six required postoperative IABP with ECMO, with four patients escalating from IABP alone during weaning from CPB. In urgent surgeries, preoperative Impella CP was used in two cases: in one case, the device was removed intraoperatively due to preserved cardiac function, while in the other case, it was continued postoperatively. One patient requiring preoperative IABP and ECMO had an LVEF of less than 30% and was suspected of having a large myocardial infarction due to a culprit lesion in the LMT. Considering the anticipated prolonged recovery of cardiac function and the potential need for extended postoperative MCS support, a decision was made intraoperatively to escalate to Impella 5.5 in combination with ECMO, followed by CABG. In both emergency (IABP: two patients) and urgent (IABP: one patient, Impella: one patient) surgeries, two patients each were successfully weaned off MCS intraoperatively. Among the elective surgeries, one patient required intraoperative IABP. Each patient was assigned to a single MCS category without overlap.

The average duration of postoperative ICU stay was 9.7 days for emergency surgeries, 6.4 days for urgent surgeries, and 2.7 days for elective surgeries. ICU stay was significantly longer in emergency cases compared to elective cases (p < 0.05). The median ICU stay was five days (interquartile range, IQR: 3.75-10.25) for emergency, four days (IQR: 3-6) for urgent, and three days (IQR: 2-3) for elective surgeries. Postoperative complications were more frequent in the emergency (13 patients, 43.3%) and urgent (five patients, 27.8%) groups, whereas no major complications occurred in the elective group (p < 0.05). The higher incidence of complications and the prolonged recovery they required may have contributed to the broader range of ICU stay durations observed in the emergency and urgent groups. Hospital mortality was recorded in one emergency surgery case (3.3%) and in one patient with CS (3.4%), with an overall mortality of one patient (1.7%). These groups were not mutually exclusive, as some emergency cases were also complicated by CS. The timing of the surgery was clearly correlated with the incidence of postoperative complications. The most common complications following emergency and urgent surgeries were sepsis and AKI. Furthermore, three patients (10%) of emergency surgeries required reexploration due to bleeding. Postoperative ECMO cases (n = 7) were associated with higher incidences of reexploration for bleeding (n = 2), gastrointestinal bleeding (n = 1), AKI (n = 3), and the need for RRT (n = 3). Preoperative echocardiography revealed reduced LVEF in both emergency and urgent surgeries (43.9% and 40.6%, respectively), whereas patients who underwent elective surgery exhibited only mild impairment. Postoperatively, LVEF improved across all surgical groups (emergency: 50.8%; urgent: 49.4%; elective: 63.6%). Notably, the proportion of patients with an LVEF of <35% was reduced by half. The intraoperative and postoperative details are summarized in Table 2.

**Table 2:** Intraoperative and postoperative details Data are presented as mean ± SD and n (%) Statistical significance among the three groups was followed by post hoc pairwise comparisons: A significant difference ^*^between Group “Elective" and Group “Emergent", and ^**^between Group “Urgent" and Group “Emergent" (p < 0.05) Each patient was assigned to a single MCS category without overlap LIMA, left internal mammary artery; LAD, left anterior descending branch; MCS, mechanical circulatory assist device; IABP, intra-aortic balloon pump; ECMO, extracorporeal membrane oxygenation; ICU, intensive care unit; DSWI, deep sternal wound infection; RRT, renal replacement therapy; GI, gastrointestinal; LVEF, left ventricular ejection fraction; SD: standard deviation

Parameter	Emergent (n = 30)	Urgent (n = 18)	Elective (n = 11)	Overall (n = 59)	p value
Japan score (%)	13.9 ± 9.7^*^	8.7 ± 9.6	4.3 ± 7.1^*^	10.5 ± 9.9	0.0006
Number of grafts	3.1 ± 1.1^*^	3.7 ± 0.9	3.9 ± 1.1^*^	3.5 ± 1.1	0.0708
LIMA to LAD	21 (70%)	18 (100%)	11 (100%)	50 (84.7%)	<0.0001
Preoperative MCS					
IABP	19 (63.3%)	9 (50%)	-	28 (47.4%)	0.0039
IABP+ECMO	2 (6.7%)	1 (5.5%)	-	3 (5.1%)	
Impella CP	-	2 (11.1%)	-	2 (3.4%)	
None	9 (30%)	6 (33.3%)	11 (100%)	26 (44.1%)	
Postoperative MCS					
IABP	13 (43.3%)	8 (44.4%)	1 (9.1%)	22 (37.2%)	0.0199
IABP+ECMO	6 (20%)	-	-	6 (10.7%)	
Impella CP	-	1 (5.6%)	-	1 (1.7%)	
Impella 5.5+ECMO	-	1 (5.6%)	-	1 (1.7%)	
None	11 (36.7%)	8 (44.4%)	10 (90.9%)	29 (49.2%)	
Postoperative course					
ICU stay (days)	9.7 ± 10.6^*^	6.4 ± 8	2.7 ± 0.8^*^	7.0 ± 8.4	0.0012
Hospital death	1 (3.3%)	-	-	1 (1.7%)	0.1351
Major complications	13 (43.3%)	5 (27.8%)	-	18 (30.1%)	0.0430
Stroke	2 (6.7%)	-	-	2 (3.4%)	0.3935
Sepsis	3 (10%)	3 (16.7%)	-	6 (10.2%)	0.3538
Pneumonia	2 (6.7%)	-	-	2 (3.4%)	0.3677
Tracheostomy	1 (3.3%)	1 (5.6%)	-	2 (3.4%)	0.7247
DSWI	2 (6.7%)	-	-	2 (3.4%)	0.3677
Acute renal injury	3 (10%)	3 (16.7%)	-	6 (10.2%)	0.3226
Newly RRT	2 (6.7%)	1 (5.6%)	-	3 (5.1%)	0.5343
Re-thoracotomy	3 (10%)	-	-	3 (5.1%)	0.3677
GI bleeding	1 (3.3%)	-	-	1 (1.7%)	0.6116
Echocardiography					
Preoperative LVEF (%)	43.9 ± 18.3^*^	40.6 ± 12.1^**^	58.2 ± 14^*,**^	45.8 ± 16.7	0.0114
Postoperative LVEF (%)	50.8 ± 15.9^*^	49.4 ± 12.1^**^	63.6 ± 9.5^*,**^	52.5 ± 14.4	0.0226
Postoperative LVEF < 35%	5 (16.7%)	2 (11.1%)	-	7 (11.9%)	0.3409

The impact of Impella on CABG for STEMI

**Figure 1:**
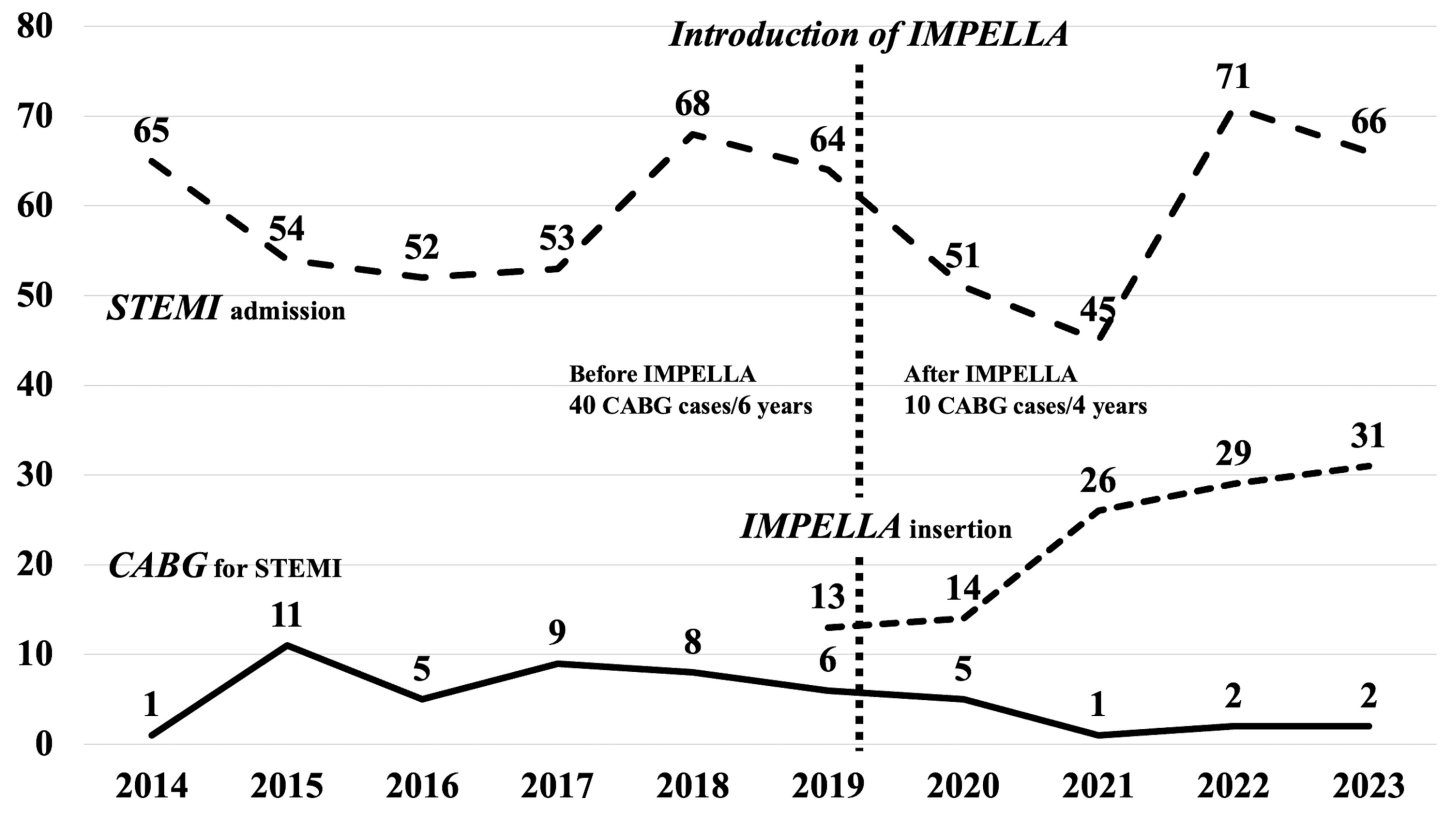
Relationship between the number of STEMI admissions, the number of Impella insertions for CS, CABG case volume for STEMI, and the introduction of Impella over the study period STEMI, ST-elevation myocardial infarction; CS, cardiogenic shock; CABG, coronary artery bypass grafting

Figure 1 illustrates the number of STEMI admissions, patients who required Impella insertion for CS of any cause, and CABG procedures performed for STEMI at our institution. Meanwhile, since the introduction of Impella in April 2019 and with the subsequent improvement in proficiency in its use, the number of CABG procedures performed for STEMI has decreased despite a consistent rate of STEMI admissions. Prior to the introduction of the Impella device, 49 CABG procedures were performed over six years, whereas only 10 were performed in the four years following its introduction. This led to a decrease in the rate of CABG performed for STEMI from 11.2% to 4.3%.

Table 3 summarizes the patient background, indications, and surgical timing of CABG before and after the introduction of Impella. No differences were observed in the severity of coronary lesions or incidence of CS. However, the indications for CABG have shifted, with an increased frequency of cases due to PCI failure. Although preoperative LVEF was lower in the unsuitable PCI group compared to the failed PCI group (41.8% ± 16.6% vs. 48.1% ± 16.9%, p < 0.05), there were no significant differences in the incidence of major complications or in-hospital mortality. Postoperative LVEF was also comparable between the groups (50.9% ± 14.6% vs. 50.3% ± 15.3%). Preoperative MCS primarily comprised IABP even after the introduction of Impella; however, the usage rate of IABP showed a decreasing trend (n = 26, 53.1% to n = 4, 40%), while Impella insertion accounted for two patients (20%). In addition, the proportion of patients requiring MCS preoperatively increased from 53.1% (n = 26) to 70% (n = 7). In-hospital mortality did not differ significantly between the periods before and after the introduction of Impella.

**Table 3:** The impact of Impella on CABG backgrounds and indications Each patient was assigned to a single MCS category without overlap LMT, left main trunk; +α, with or without other vessel disease; CABG, coronary artery bypass grafting; PCI, percutaneous coronary intervention; MCS, mechanical circulatory assist device; IABP, intra-aortic balloon pumping; ECMO, extracorporeal membrane oxygenation

Parameter	Before Impella (n = 49), n (%)	After Impella (n = 10), n (%)	p value
Coronary angiography			
LMT+α	23 (46.9%)	3 (30%)	0.5403
Three-vessel disease	18 (36.7%)	4 (40%)	
Two-vessel disease	7 (14.3%)	3 (30%)	
One-vessel disease	1 (2%)	-	
Indications of CABG			
Unsuitable PCI	37 (75.5%)	6 (60%)	0.5678
Failed PCI	4 (8.2%)	3 (30%)	
Post-PCI residual lesions	6 (12.2%)	1 (10%)	
PCI complications	1 (2%)	-	
Coexisting valvular disease	1 (2%)	-	
Cardiogenic shock	24 (49%)	5 (50%)	0.5727
Operative status			
Emergent	26 (53.1%)	4 (40%)	0.4658
Urgent	13 (26.5%)	5 (50%)	
Elective	10 (20.4%)	1 (10%)	
Preoperative MCS			
IABP	24 (48.9%)	4 (40%)	0.0602
IABP+ECMO	2 (4.1%)	1 (10%)	
IMPELLA CP	-	2 (20%)	
None	23 (46.9%)	3 (30%)	
Postoperative MCS			
IABP	19 (38.8%)	3(40%)	0.0982
IABP+ECMO	5 (10.2%)	1 (10%)	
Impella CP	-	1 (10%)	
Impella 5.5+ECMO	-	1 (10%)	
None	25 (51%)	4 (30%)	

## Discussion

This study demonstrated favorable outcomes for CABG in patients with STEMI, with an overall in-hospital mortality rate of 1.7%. Mortality was 3.3% among patients who underwent emergency surgery and 3.4% among those presenting with CS. However, surgical timing was strongly correlated with the incidence of postoperative complications and the length of ICU stay, highlighting the importance of careful perioperative management.

To contextualize temporal changes, we compared outcomes and MCS utilization before and after the introduction of the Impella device. Although there was no statistically significant difference in overall in-hospital mortality between the two periods, the rate of preoperative MCS use increased from 53.1% to 70%, with Impella accounting for 20% of cases after its introduction. Notably, the implementation of Impella appeared to shift CABG indications from cases deemed unsuitable for PCI toward those involving failed PCI. As a result, although the overall number of CABG procedures declined, the proportion of urgent surgeries increased, while elective CABG procedures became less frequent.

CABG for STEMI

The 30-day mortality rate for CABG in AMI is reported to range from 3% to 8%, with contributing risk factors including cardiac arrest, shock, advanced age, female sex, diabetes, low LVEF, renal dysfunction, and intervention within 24 hours [5,6,8,19-21]. For patients experiencing CS, mortality has increased to approximately 19% [19,22], with no significant improvement since the early 2000s [22]. In contrast, CABG for hemodynamically stable patients with STEMI within 48 hours has shown favorable outcomes, resulting in a 30-day mortality rate of 2.7%. This is typically due to the fact that these procedures are performed in younger patients with fewer comorbidities [7]. These findings highlight that maintaining stable hemodynamics while ensuring myocardial and major organ protection is essential for improving the outcomes of CABG in patients with STEMI.

Although this study showed a trend toward higher predicted 30-day mortality rates with increasing surgical urgency, our actual surgical outcomes were more favorable than both the predicted 30-day mortality and previously reported rates. The overall mortality rate was 1.7%, with 3.3% for emergency surgery and 3.4% for CS. Several factors may explain this lower mortality rate.

First, our standard use of on-pump arrest CABG in patients with STEMI before the introduction of Impella likely contributed to improved outcomes. This approach enables complete revascularization even in hemodynamically unstable patients with low LVEF or CS, thereby enhancing the function of the viable myocardium. Myocardial protection was achieved through cardioplegia, myocardial cooling, and LV decompression, which helped salvage the ischemic myocardium. In addition, using CPB ensures perfusion to the major organs during surgery, which may further improve patient outcomes.

Second, a proactive MCS strategy plays a critical role. Previous studies have demonstrated that surgical mortality in CABG for AMI-CS varies based on the timing of MCS initiation: 16% in patients without preoperative MCS, 37.2% in those requiring preoperative MCS, and 58.4% in those who did not receive preoperative MCS but required it intraoperatively or postoperatively [22]. Furthermore, in CABG for patients with low LVEF experiencing postcardiotomy CS, early intraoperative placement of Impella 5.0 or 5.5 resulted in a 30-day survival rate of 75.6%, compared with 47.6% when MCS was introduced later postoperatively [23]. These findings underscore the importance of early hemodynamic support and LV unloading in high-risk patients undergoing CABG.

In our cohort, 83% of patients with CS received preoperative MCS, with 16.7% undergoing escalation during surgery. Overall, 87.5% of patients remained on MCS postoperatively, reflecting an aggressive approach aimed at facilitating safe CPB weaning and ensuring optimal postoperative hemodynamic support. Maintaining stability through appropriate circulatory support, both pre- and postoperatively, is crucial for myocardial recovery and improved patient outcomes. Following the introduction of Impella, there has been a trend toward increased pre- and postoperative MCS utilization. This may reflect a growing awareness and understanding of appropriate MCS strategies in the management of CS.

Despite favorable outcomes for CABG overall, emergency and urgent surgeries continue to be associated with high rates of postoperative complications, including AKI, bleeding, and infections, which often lead to prolonged ICU stays. Moreover, critical surgical factors such as the rate of LIMA-to-LAD grafting and the number of bypass grafts tend to be lower in emergency cases, potentially compromising long-term outcomes. In our cohort, 16.7% of patients initially supported with preoperative IABP required intraoperative escalation to IABP plus ECMO. Furthermore, patients requiring postoperative ECMO support experienced higher rates of major complications, including reexploration for bleeding, gastrointestinal bleeding, AKI, and the need for RRT. These findings highlight the limitations of conventional support strategies such as IABP and ECMO in managing the most critically ill patients.

Therefore, to improve both acute and long-term outcomes in high-risk STEMI patients, particularly those with CS, there is a need to explore optimized MCS strategies beyond IABP and ECMO. Such strategies should aim to protect ischemic myocardium, support myocardial recovery, preserve major organ function, and allow for better control over the timing of surgical revascularization.

Impact of Impella on CABG for STEMI

Impella improves PCI outcomes in high-risk cases, including AMI-CS, low ejection fraction with MVD, and LMTD [14,18]. In the Japan Registry for Percutaneous Ventricular Assist Device (J-PVAD) registry, Impella-assisted PCI was performed in 56.5% of AMI-CS cases, with coronary lesions involving the LMTD in 37% and MVD in 73.7% [16]. These findings suggest that Impella support facilitates high-risk PCI and influences treatment strategies for STEMI. At our institution, the introduction of Impella has shifted the indications for CABG in STEMI from PCI ineligibility to PCI failure, leading to a decrease in the rate of CABG for STEMI. Advances in PCI techniques and MCS have made high-risk PCI safer, which is a positive development; however, several concerns remain.

First, although CABG indications for STEMI are shifting toward PCI failure in the era of Impella, previous studies have reported increased hospital mortality and adverse cardiovascular events when CABG was performed after failed PCI. Thielmann et al. reported that the hospital mortality rate for CABG performed within 24 hours of PCI for STEMI was 15.8%, rising to 21.1% following failed PCI, with an in-hospital cardiovascular event rate of 31.1% [24]. Patients with prior PCI also had a significantly higher rate of re-thoracotomy than those without prior PCI (8.7% vs. 5.7%, p = 0.003), indicating an increased risk of postoperative bleeding. Furthermore, the need for postoperative IABP (31% vs. 15%, p < 0.001) and ECMO (4% vs. 2%, p < 0.001) was significantly higher in patients with failed PCI, reflecting greater hemodynamic instability in this group [24]. Further investigations are required to determine whether these trends persist in the current era of Impella support.

Another critical concern is the need for incomplete revascularization. CABG has shown superior outcomes compared to PCI in patients with AMI due to MVD, particularly in those experiencing CS, where approximately 50% of patients have MVD. The advantage is largely due to the ability to achieve complete revascularization [4,19,20]. Incomplete revascularization in AMI negatively affects both short- and long-term outcomes [25,26]. Although the PROTECT III study demonstrated that Impella support improves complete revascularization rates in high-risk PCI procedures [18], only 66.3% of patients with AMI-CS achieved complete revascularization in the J-PVAD registry [16].

Despite its potential benefits, the implementation rate of CABG in patients with AMI and MVD remains low. In one study, although the heart team recommended CABG for 30% of patients with STEMI and residual nonculprit lesions, only 1.4% ultimately underwent surgery [26]. Similarly, in the J-PVAD registry, CABG was performed in only 2.5% of cases [16], with reports suggesting CABG rates for AMI-CS range from 3% to 8% [19]. Several factors contribute to this reluctance, including the increased bleeding risk associated with dual antiplatelet therapy (DAPT) initiation after PCI, the risk of acute thrombosis with DAPT discontinuation for surgery, and the historically poor outcomes of CABG in high-risk STEMI cases [26].

However, the introduction of Impella may help overcome these challenges. In some patients, the Impella placement alone restores perfusion to the culprit lesion and halts ischemic progression. These findings suggest that immediate PCI may not always be necessary, particularly in patients with extensive coronary disease. In such cases, deferring or avoiding PCI may also allow clinicians to withhold DAPT, which can be advantageous in surgical candidates or those at high risk of bleeding.

For patients in whom CABG is the preferred revascularization strategy but has historically been avoided due to surgical risk, Impella offers a paradigm shift. By ensuring ischemia cessation, myocardial protection, recovery from CS, and major organ preservation, Impella facilitates safer and well-timed CABG, allowing complete and durable revascularization in high-risk patients with STEMI. These findings suggest that in the era of Impella, CABG may regain a more prominent role in STEMI management, offering an alternative to PCI in selected high-risk cases.

We have transitioned from performing on-pump arrested CABG to Impella-assisted beating CABG for patients with STEMI having CS. This shift was driven by the recognition that Impella support not only facilitates complete revascularization during CABG by maintaining hemodynamic stability but also enables its use in even hemodynamically unstable STEMI cases. By avoiding CPB, this approach helps mitigate CPB-associated complications such as systemic inflammatory response and coagulopathy. In addition, Impella provides continuous myocardial and organ protection throughout the perioperative period, further enhancing the safety of surgical revascularization and improving postoperative outcomes.

However, to safely perform beating-heart CABG under Impella support, specific surgical expertise is required. Close intraoperative coordination with anesthesiologists and clinical engineers is also essential to ensure appropriate device management and hemodynamic monitoring. Moreover, in patients with poor oxygenation or concomitant right heart failure, achieving hemodynamic stability during surgery may be particularly challenging, and careful perioperative planning is warranted.

Although reports on Impella-assisted CABG for STEMI exist [27], newer treatment strategies have emerged, including the staged beating of the CABG following PCI under Impella support, with the escalation of MCS as needed [28]. However, the current evidence remains limited, underscoring the need for further clinical studies to refine patient selection and optimize outcomes. In addition, the potential risks associated with Impella, such as bleeding, limb ischemia, hemolysis, and the need for RRT, must be carefully weighed during treatment planning [14]. Beyond clinical risks, future research should also assess the economic burden and impact on ICU resource utilization associated with Impella-supported strategies to better evaluate their feasibility and sustainability in broader clinical practice.

Nevertheless, the advent of Impella is a significant treatment strategy for STEMI. Moving forward, the heart team needs to develop new treatment paradigms that integrate emerging device technologies and optimize surgical decision-making during the IMPELLA era.

Study limitations

This study has several limitations. It was a single-center, retrospective analysis with a small sample size, which may affect the generalizability of the findings and limit statistical power. The selection and timing of CABG were determined by the heart team on a case-by-case basis, introducing potential selection bias in treatment allocation. Moreover, unmeasured confounding factors, such as baseline comorbidities, infarct size, and completeness of revascularization, may have influenced outcomes and limited the interpretation of results. The relatively short interval between Impella insertion and CABG, as well as the limited number of CABG procedures performed after Impella adoption, requires continued observation to assess evolving trends. In addition, outcomes were evaluated only during hospitalization, and long-term prognostic data were not included. Finally, the small sample size and retrospective design may have contributed to the underreporting of postoperative complications.

## Conclusions

In conclusion, this study suggests that CABG in patients with STEMI, particularly those experiencing CS, can be associated with favorable outcomes when supported by preoperative MCS, including the use of the Impella device. Our findings indicate a potential association between Impella use and a shift in CABG indications, from unsuitable PCI to failed PCI, along with a trend toward reduced emergency procedures and increased urgent surgeries requiring careful hemodynamic management and timing.

While CABG remains an essential strategy for selected high-risk STEMI patients, these results should be interpreted with caution given the retrospective, single-center design. Further multicenter, prospective studies are warranted to validate these findings and minimize institutional bias. Future efforts should focus on optimizing surgical timing, refining patient selection criteria for MCS use, and integrating device-based strategies into standardized heart team decision-making. Additionally, considerations such as cost and accessibility may affect the broader applicability of Impella-supported approaches and should be addressed in future investigations.
